# The Defective and Dependent


**Published:** 1912-02-10

**Authors:** 


### THE DEFECTIVE AND DEPENDENT.*

### V.—The Education of the Blind.

REFERENCE has already been made in a previous paper (December 2, 1911, page 181) to the early history of the training of the blind. We now propose to consider in further detail the actual methods of instruction of blind children which, since the Act of 1893, have formed a part of our national system of education.

It will be helpful to an understanding of the varied capacities of blind pupils to consider briefly the chief causes of the affliction which necessitates their being treated educationally as a class apart. Following the ætiological grouping laid down by Dr. N. B. Harman, ophthalmologist to the London County Council Blind Schools, in an interesting paper read before the International Congress of School Hygiene in London in 1907, we find that in the Council schools accidental causes account for some 44 per cent. of the cases, including 37 per cent. due to ophthalmia neonatorum. This is an entirely preventible cause, and happily, owing to increased hygienic precautions enforced by the Central Midwives Board, seems to be in a fair way of being prevented. Purulent ophthalmia at later ages accounts for 7 per cent. of the group, and a similar proportion of cases is due to accidental injuries and sympathetic inflammation of the eyes. Between 20 and 30 per cent. of the cases appear to be due to congenital syphilis; about 20 per cent. suffer from congenital deformities from arrested development affecting the eye; and there is a further small group of eye defects, such as high myopia, which, though not causing absolute blindness, produces incapacity for benefiting by the ordinary visual means of education.

Those blind by reason of accidental causes are the most promising from the educational point of view, inasmuch as the intellectual faculties are normal; the group of syphilitic cases tend to degeneration of sense organs and brain-cells, and those suffering from developmental defects of the eyes are apt to be handicapped by concomitant physical or mental imperfections. A knowledge of these physiological groups is obviously essential to correct classification for purposes of instruction, and points to the importance of expert medical co-operation in schools for the blind.

The deprivation of vision cuts off the blind child from the mental stimulus of its surroundings, and with " knowledge at one entrance quite shut out " a compensatory training of the mind through the other senses has to be arranged. Oral instruction conveyed in an interesting manner by tactful, discriminating, and loving teachers is, of course, the first essential, and success depends, not only upon the subjects taught but the manner in which they are presented, so as to develop the child's own powers of observation and perception, and to train the reasoning faculties.

The opening up of a path to the brain through the fingers is the next problem that has to be dealt with. The current belief that the blind child has naturally a more delicate tactile sense than the sighted child does not appear to be founded on fact, and exercises in the digital perception of surface contrasts, such as rough and smooth, resisting and yielding, etc., have to be sedulously employed. By degrees notions of extension in space and of form and size are tactually developed, and in the more progressive schools a course of adapted kindergarten exercises for the younger children precedes the study of the " 3 R's." Reading is now almost universally taught on the system invented in 1829 by Louis Braille (himself blind), which consists of varying combinations of six raised points placed in an oblong, of which the vertical side may comprise three and the horizontal two points. The letter A, for example, is represented thus:—

A, ● . – B, ● . – C, ● ●
. .      ● .      . .
. .      . .      . .

and so on. There are also certain similar symbols for short words. Similar characters are used in writing, which is kept regular by means of a frame furnished with a brass guide. Musical notation is also arranged by means of Braille characters, which indicate the lines and spaces, value of the notes, and other signs. For teaching arithmetic ciphering-boards are used perforated by star-shaped openings into which square pins are placed, indicating by their respective positions with regard to the points of the star the different figures. Relief maps are used for the teaching of geography.

Literally accomplishments such as those described above should, however, be regarded as in a sense supplemental to a sound basis of physical and manual training. Most blind children have a comparatively poor physique and are timid, awkward, and listless, so that it is essential to educational success that their muscular powers should be developed, both by gymnastic exercises and games, and self-dependence fostered as much as possible. In such an institution as the Royal Normal College for the Blind at Norwood the pupils are trained to a wonderful pitch of perfection in a variety of athletic exercises and many become expert swimmers, cyclists, roller-skaters, and even oarsmen. Music, both instrumental and vocal, is professionally taught. Manual training carried on from the kindergarten stage to the use of the hammer, saw, and plane leads up to practical industrial work, such as pianoforte tuning and typewriting, whilst among the ordinary industries practised by the blind may be mentioned basket, brush, mattress and sack making, upholstery of various kinds, chair-caning, knitting, and hand and machine sewing.

We propose to consider the subject of industrial training and its results in a subsequent article.

* Previous articles in this series have appeared in THE HOSPITAL of Nov. 4, Nov. 18, Dec. 2, and Jan. 6.

